# Enhanced IL-6 and IL-12B Gene Expression After SARS-CoV-2 Infection in Leprosy Patients May Increase the Risk of Neural Damage

**DOI:** 10.4269/ajtmh.21-0034

**Published:** 2021-04-05

**Authors:** Gilberto Santos Morais Junior, Patrícia Shu Kurizky, Selma Regina Penha Silva Cerqueira, Daniel Holanda Barroso, Heidi Luise Schulte, Cleandro Pires de Albuquerque, Eliana Teles de Gois, Laila Salmen Espindola, Jaime Martins Santana, Izabela Marques Dourado Bastos, Carla Nunes de Araújo, Licia Maria Henrique da Mota, Otávio Toledo Nóbrega, Ciro Martins Gomes

**Affiliations:** 1 Programa de Pós-Graduação em Ciências da Saúde, Faculdade de Ciências da Saúde, Universidade de Brasília (UnB) Brasília, Brazil;; 2 Programa de Pós-Graduação em Ciências Médicas, Faculdade Medicina, Universidade de Brasília (UnB) Brasília, Brazil;; 3 Hospital Universitário de Brasília (HUB), Brasília, Brazil;; 4 Instituto de Ciências Biológicas, Universidade de Brasília (UnB) Brasília, Brazil;; 5 Programa de Pós-Graduação em Medicina Tropical, Núcleo de Medicina Tropical, Faculdade Medicina, Universidade de Brasília (UnB) Brasília, Brazil

## Abstract

Experts have called attention to the possible negative impact of the coronavirus disease 2019 (COVID-19)–related cytokine storm syndrome on the progression of leprosy-related disabilities. We assessed the frequency of reactional states in patients co-infected with *Mycobacterium leprae* and severe acute respiratory syndrome (SARS) coronavirus (CoV) 2 (SARS-CoV-2). We consecutively included patients during the first peak of the COVID-19 epidemic in Brazil and analyzed the expressions of genes encoding interleukin (IL)-1β, IL-6, IL-8, IL-10, IL-12A, IL-12B, and tumor necrosis factor-α in peripheral blood mononuclear cells. We included 64 leprosy patients and 50 controls. Twelve of the leprosy patients and 14 of the controls had been diagnosed with COVID-19. Co-infection was associated with increased IL-6 (*P* = 0.043) and IL-12B (*P* = 0.017) expression. The median disability grades were higher for leprosy/COVID-19 patients; however, the difference was not significant (*P* = 0.194). Patients co-infected with *M. leprae* and SARS-CoV-2 may experience a higher-grade proinflammatory state.

The outbreak of the new coronavirus disease 2019 (COVID-19) has alarmed healthcare professionals. The increased mortality rates associated with COVID-19 are a result of hypercytokinemia, which is also referred to as cytokine storm syndrome.^1,2^ Despite global efforts to contain the spread of COVID-19, it has reached all continents.^1^ Leprosy is a chronic infectious disease caused by *Mycobacterium leprae* that is still endemic in highly populated countries such as Brazil.^3^ Despite its infectious nature, the disease has an important autoimmune background. Soluble factors derived from the bacillus can stimulate immune reactions, ultimately resulting in acute clinical pictures called leprosy reactional states.^4,5^

The onset of the COVID-19 pandemic has hindered the proper management of leprosy patients because the social distancing strategies may act as an important barrier to accessing polychemotherapy. Recently, experts in the field called attention to the possible negative impacts of cytokine storm syndrome on the progression of leprosy.^6,7^ As seen with other infections, severe acute respiratory syndrome (SARS) coronavirus (CoV) 2 (SARS-CoV-2) infection in patients with leprosy may stimulate refractory reactional states.

We aimed to assess the frequency of reactional states in leprosy patients after recovery from COVID-19 and to measure the impact of *M. leprae* and SARS-CoV-2 co-infection on the immunological response by measuring gene expressions in a controlled clinical model.

We performed a cross-sectional study and consecutively included patients attending the Leprosy Ambulatory Clinic of the University Hospital of Brasília, Brazil, from April to October 2020, during the first peak of the COVID-19 outbreak in midwestern Brazil. The professionals at this ambulatory clinic are responsible for diagnosing cases of leprosy and performing differential diagnoses. We excluded any immunosuppressed patients other than those using prednisone for the treatment of leprosy reactions. The effects of the duration of the first symptoms of COVID-19 and other relevant variables were measured, including the occurrence of leprosy reactional states after COVID-19.

To assess the association of gene expression with cytokine levels, blood was obtained from each patient and tumor necrosis factor (TNF)-α, interleukin (IL)-10, IL-1β, IL-8, IL-6 and IL-12p70 were measured in the plasma using the Human Inflammatory Cytokine Cytometric Bead Array - I Kit (RUO) (Becton Dickinson, Franklin Lakes, NJ) in a FACSVerse flow cytometer (Becton Dickinson). Peripheral blood mononuclear cells (PBMCs) were isolated using Ficoll^®^ Paque Plus (GE Healthcare Bio-Sciences AB, Uppsala, Sweden) gradient centrifugation. High-molecular-weight RNA was purified using the mirVANA Paris Kit (Thermo Fisher Scientific, Waltham, MA) with 700 µL of PBMCs. RNA was quantified spectrophotometrically (NanoDrop 2000/2000c; Thermo Fisher Scientific). Then, 100 ng of cDNA was prepared using the High-Capacity RNA Reverse Transcription kit (Thermo Fisher Scientific). Gene expression assays were performed for the immune mediators TNF-α, IL-10, IL-1β, IL-8, IL-6, IL-12A, and IL-12B using quantitative polymerase chain reaction (PCR) with TaqMan-specific reagents (Thermo Fisher Scientific) on QuantStudio 1 (Thermo Fisher Scientific) and standard settings. Relative quantification was based on a β-globulin target using the 2^−ΔΔCq^ method.^8^

Data analysis was performed with statistical software R version 4.0.3 (R Foundation for Statistical Computing, Vienna, Austria). The final model consisted of a multivariable linear regression in which cytokine gene expression values were considered the dependent variables and clinically relevant variables that could influence gene expression levels (COVID-19 diagnosis, leprosy diagnosis, and the use of prednisone or an equivalent at a daily dosage of 1 mg/kg/day after COVID-19) were considered the independent variables. *P* < 0.05 indicated that the independent variable significantly influenced cytokine expression. All patients were included after signing an informed consent form. This study was approved by Brazil’s National Committee of Ethics (CAAE 34164820.6.0000.0030).

We included 114 consecutive patients; 64 were leprosy patients (before, during, and up to 3 years after the end of polychemotherapy for leprosy) and 50 were controls (who attended the leprosy ambulatory clinic and were not diagnosed with leprosy). Twelve leprosy patients (mean time since the onset of COVID-19, 43.54 days; range, 6-75 days) and 14 nonleprosy patients (mean time since the onset of COVID-19, 41.84 days; range, 6-90 days) reported having been diagnosed with COVID-19 based on reverse-transcriptase PCR amplification of SARS-CoV-2 RNA from upper airway samples. The demographic characteristics of each group are shown in Table 1. No patient needed intensive care support; therefore, it was not possible to compare patients with severe cases of COVID-19 to those with mild cases. The time from the onset of COVID-19 until inclusion was not different between the two groups (*P* = 0.899). The levels of the tested cytokines were not related to the time from the onset of COVID-19 to inclusion. The number of previous bacille Calmette-Guerin (BCG) vaccine doses was not related to the gene expression profiles. No patients reported having used medications intended to have a suppressive effect on SARS-CoV-2. According to the institutional protocol, when leprosy patients using high doses of corticosteroids were diagnosed with COVID-19, the daily doses were reduced to 20 to 40 mg of prednisone for 15 days.

**Table 1:** Fold-changes in the gene expression of TNF-α, IL-10, IL-1β, IL-8, IL-6, IL-12, and IL-12B and the adjusted effects of COVID-19, leprosy, and reactional states according to the multivariate linear regression model

Demographic characteristics	Leprosy/COVID-19	COVID-19	Leprosy	Controls	*P* value
Sex					0.482
Male, n (%)	7 (58.3%)	4 (28.6%)	24 (46.2%)	17 (47.2%)	
Female, n (%)	5 (41.7%)	10 (71.4%)	28 (53.8%)	19 (52.8)	
Age, years, mean (SD)	52.6 (11.2)	37.6 (16.3)	42.2 (13.9)	42.94 (15.63)	0.033
Type I reaction, n (%)	3 (25.0%)	–	20 (38.5%)	–	0.411
Type II reaction, n (%)	1 (8.3%)	–	9 (17.3%)	–	0.500
Disability grade, median (IQR)	2 (1)	–	1 (1)	–	0.194
Time since the onset of COVID-19, days, mean (SD)	43.5 (19.9)	41.8 (26.8)	–	–	0.899
Multibacillary leprosy	10 (83.3%)		42 (80.8%)		0.885
Gene expression	^2ΔΔ^Cq (SD)				*P* value*
TNF-α	11.1 × 10^5^ (38.4 × 10^5^)	15.8 × 10^2^ (51.4 × 10^2^)	10.5 × 10^3^ (38.1 × 10^3^)	598.46 (23.1 × 10^2^)	0.061
IL-10	13.9 × 10^4^ (48.1 × 10^4^)	28.2 × 10^2^ (64.6 × 10^2^)	14.4 × 10^2^ (36.5 × 10^2^)	45.9 × 10^1^ (17.5 × 10^2^)	0.066
IL-1β	43.1 × 10^5^ (49.4 × 10^5^)	79.6 × 10^1^ (14.7 × 10^2^)	95.2 × 10^2^ (37.7 × 10^3^)	46.2 × 10^1^ (17.8 × 10^2^)	0.066
IL-8	36.1 × 10^5^ (12.5 × 10^6^)	13.9 × 10^2^ (36.4 × 10^2^)	20.5 × 10^3^ (10.5 × 10^4^)	45.1 × 10^1^ (14.8 × 10^2^)	0.066
IL-6	11.8 × 10^10^ (4.1 × 10^10^)	49.4 × 10^1^ (1.3 × 10^3^)	60.8 × 10^2^ (2.0 × 10^4^)	19.3 × 10^1^ (6.7 × 10^2^)	0.046
IL-12A	19.2 × 10^5^ (66.5 × 10^5^)	82.7 × 10^6^ (30.9 × 10^7^)	84.5 × 10^2^ (48.9 × 10^3^)	12.7 × 10^2^ (64.2 × 10^2^)	0.512
IL-12B	17.0 × 10^4^ (48.4 × 10^4^)	31.5 × 10^2^ (63.3 × 10^2^)	37.3 × 10^2^ (25,342.95)	97.1 × 10^1^ (3,886.18)	0.020

TNF = tumor necrosis factor; IL = interleukin; COVID-19 = coronavirus disease 2019; SD = standard deviation; IQR = interquartile range.

* *P* values were adjusted with a multivariate linear regression model and represent the influence of COVID-19 on the gene expression levels. *P* < 0.05 indicates that COVID-19 significantly influenced the levels of cytokine expression.

We classified patients with paucibacillary and multibacillary leprosy based on the Ridley and Joplin criteria, but the leprosy classification was not related to gene expression. The frequencies of leprosy type I and type II reactions were similar between patients with (33.3%) and without (42.30%) SARS-CoV co-infection (*P* = 0.896). Absolute gene expression levels of almost all tested cytokines were higher in *M. leprae*/SARS-CoV-2 co-infected patients (Figures 1 and 2, Table 1). The median disability grade was higher for *M. leprae*/SARS-CoV-2 co-infected patients (median, 2; interquartile range [IQR], 1) than for leprosy patients (median, 1; IQR, 1), but the difference was not significant (*P* = 0.194). Our multivariate model showed that COVID-19 was related to increased expression levels of TNF-α, IL-1β, IL-6, IL-8, IL-12B, and IL-10 (*P* < 0.05) (Table 1), and that leprosy simultaneously enhanced the levels of IL-6 (*P* = 0.046) and IL-12B (*P* = 0.020) (Figure 1). The use of prednisone reduced the level of IL-12B (*P* = 0.038). Gene expression analyses and flow cytometry yielded consistent results.

**Figure 1.**
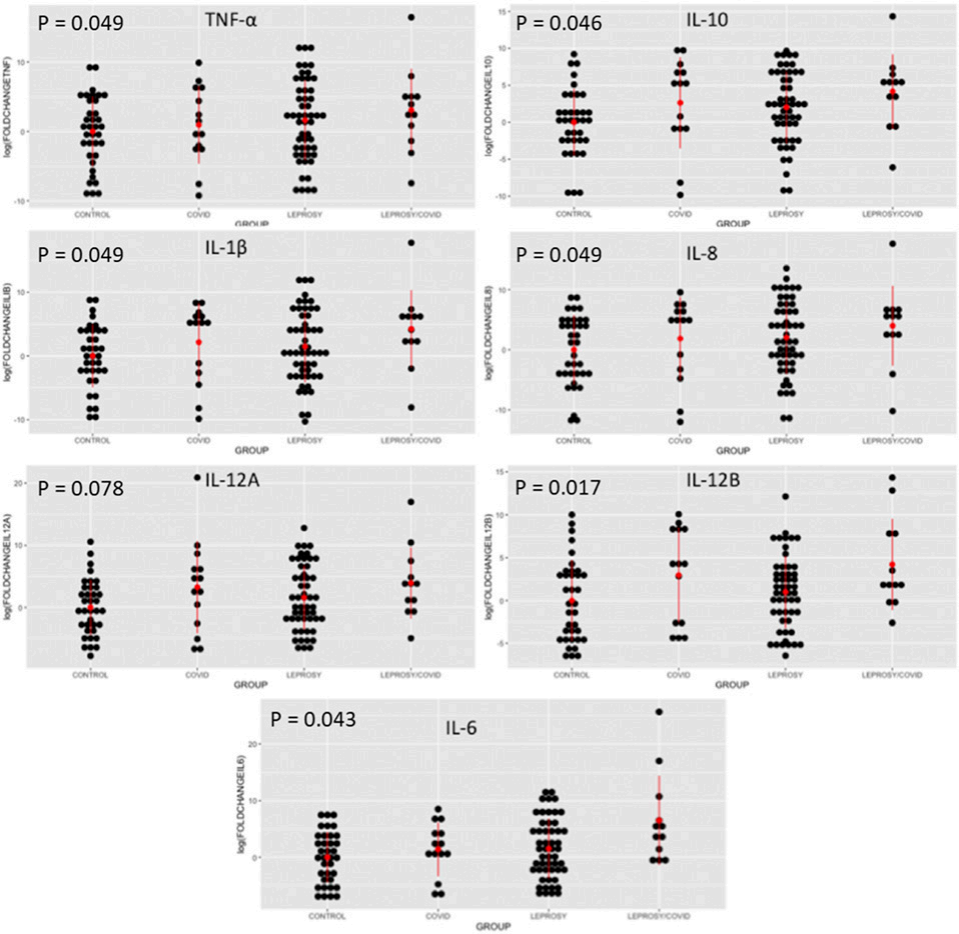
A dot plot of the four main groups included in the study. Group 1 includes patients co-infected with *Mycobacterium leprae* and SARS-CoV-2. Group 2 includes patients infected with SARS-CoV-2 but not *M. leprae*. Group 3 includes patients infected with *M. leprae* but not SARS-CoV-2. Group 4 includes control patients who were not infected with either *M. leprae* or SARS-CoV-2. *P* values were adjusted with a multivariate linear regression model and represent the influence of leprosy on the gene expression levels. *P* < 0.05 indicates that leprosy significantly influenced the levels of cytokine expression. This figure appears at www.ajtmh.org.

**Figure 2.**
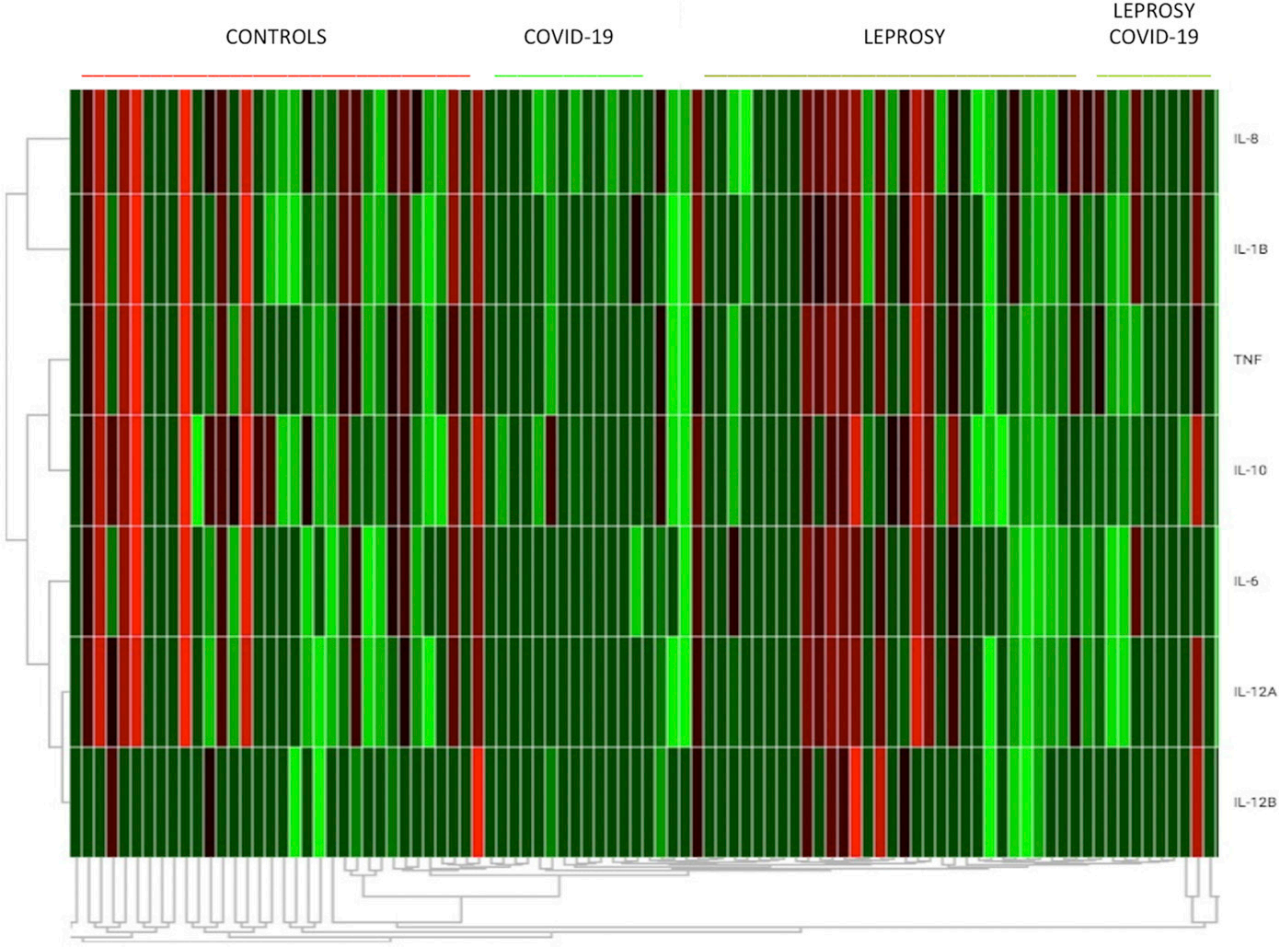
A heatmap showing increased levels of most measured cytokines in patients who had contracted coronavirus disease 2019 (COVID-19). The plot was generated using the Thermo Fisher Scientific Cloud Environment (Thermo Fisher Scientific, Waltham, MA). This figure appears at www.ajtmh.org.

As expected, our study showed that most of the cytokine levels measured were higher in patients who had contracted COVID-19 than in leprosy patients and controls, even at more than 30 days after the onset of COVID-19. The proinflammatory cytokines TNF-α, IL-1β, IL-8, IL-6, and IL12-B (*P* < 0.05) were increased in patients who had contracted COVID-19. This result indicates a possible role of COVID-19 in inflammation that could stimulate leprosy reactional states.^2,6^

The frequency of reactional states in patients co-infected with *M. leprae* and SARS-CoV-2 was not greater than that in patients with leprosy alone. However, clinicians must be aware that most parameters for the detection of leprosy reactions rely on acute clinical signs of neurological pain, and that silent neuropathy in leprosy is difficult to diagnose. This may explain why the median disability grade was higher for leprosy patients who contracted COVID-19. As an important example, TNF-α expression is related to intense phagocytic activity, which is also observed in type I leprosy reactions. IL-8 and IL-1β are related to neutrophil recruitment and are frequently detected in the skin of leprosy patients experiencing type I reactions.^9^

The expression of IL-6 was significantly increased in leprosy/COVID-19 during our multivariate analysis. A recent systematic review of the literature found increased levels of IL-6 in patients with COVID-19.^10^ Clinical studies have shown that single nucleotide polymorphisms in IL-6 genes are associated with leprosy reactions.^11^ In addition, IL-6 is a marker of neuropathic pain in leprosy, and patients with *M. leprae/*SARS-CoV-2 co-infections may be at a greater risk for silent neuropathy.^12^ This interesting result suggests the need for investigations of the use of IL-6 inhibitors, such as tocilizumab, to prevent neural damage.

Interestingly, our multivariate model also showed that IL-12B gene expression was increased in patients with *M. leprae*/SARS-CoV-2 co-infections. This cytokine is related to a cascade of Th1 cellular responses^13^ also found in leprosy type I reactions; however, its role in the pathogenesis of COVID-19 is uncertain. IL-12B is closely related to the cellular response to mycobacterial infections,^14^ and its elevation as a result of *M. leprae*/SARS-CoV-2 co-infection introduces important questions. Some experts in the field of infectious diseases have hypothesized that patients living in countries with universal BCG vaccination could have a reduced likelihood of contracting COVID-19.^15^ Could a constantly stimulated Th1 profile in patients with leprosy and their close contacts who have undergone BCG vaccination reinforce this hypothesis?

One limitation of this study was that we were unable to test other cytokines that are important in the pathogenesis of leprosy reactions, including interferon-γ. We were also unable to evaluate longitudinal changes throughout the course of COVID-19. Cross-sectional methodology is useful for raising questions; however, further longitudinal studies are needed to test the real effects of this co-infection on the generation of neuropathy.

We can conclude that *M. leprae*/SARS-CoV-2 co-infected patients experience a prolonged inflammatory state after viral infection. We were also able to observe that IL-6 and IL-12B levels were increased in *M. leprae*/SARS-CoV-2 co-infected patients, but clinically evident neuritis was not more common in those patients. Therefore, clinicians must be vigilant for the possibility of silent neuropathy in patients co-infected with *M. leprae* and SARS-CoV-2, especially those with critical cases of COVID-19.
